# Periodic fever, aphthous stomatitis, pharyngitis, and cervical adenitis syndrome (PFAPA) or recurrent urinary tract infections: a case report

**DOI:** 10.1186/s12887-021-03075-3

**Published:** 2022-01-26

**Authors:** Banafshe Dormanesh, Maryam Asli, Roya Daryanavard, Peyman Arasteh

**Affiliations:** 1 Department of Pediatric, AJA University of medical sciences, Tehran, Iran; 2 Infectious Diseases and Tropical Medicine Research Center, Tehran University of Medical Sciences, Tehran, Iran; 3 Shiraz University of Medical Sciences, Shiraz, Iran

**Keywords:** Urinary tract infection, Fever, Pediatric, PFAPA

## Abstract

**Background:**

Fever is the most frequent reason for medical consultation in children, and makes up 15–25% of all consultations in primary care and emergency departments. In here we report a case of a 13 year-old girl who referred with an unusual presentation of fever and was misdiagnosed with recurrent urinary tract infection for 8 years.

**Case presentation:**

This is a Clinical Reasoning Cycle case study. A 13 year-old girl was referred with a chief complaint of recurrent fevers from 8 years. During her first febrile episode, she had a 5-day high-grade fever associated with loss of appetite. Her physical examination at that time was unremarkable. Blood tests showed leukocytosis with a shift to the left and urine examination was in favor of pyuria. The urine culture was positive for bacterial growth. The episodes of fever were repeated every 45 days. Accordingly, the patient was diagnosed as a case of recurrent urinary tract infection. In the intervals between her febrile episodes, the patient was healthy and laboratory tests were normal. Ultrasonography, voiding cystourethrogram and dimercaptosuccinic acid scans were normal. During her last visit, the patient mentioned difficulty in swallowing and on examination cervical lymph nodes, exudative tonsillitis and painful aphthous stomatitis were detected. All antibiotics were stopped and corticosteroids were started. The patient’s symptoms were relieved and the interval between her febrile episodes became longer.

**Conclusions:**

Our study shows that a patient should never be marked, particularly when the symptom and signs aren’t completely justifying a patient’s condition.

## Background

Fever is the most frequent reason for medical consultation in children, and makes up 15–25% of all consultations in primary care and emergency departments [1]. Recurrent fevers may be due to infectious or non-infectious causes. Urinary tract infection (UTI) is among the most common infections in children [1]. Diagnosis of UTI in children can be challenging as it presents with variable and sometimes vague clinical signs and symptoms, and in some cases only fever may be the presenting sign [2].

In here we report a case of a 13 year-old girl who referred with an unusual presentation of fever and was misdiagnosed with recurrent UTI for the last 8 years.

## Case presentation

A 13 year-old girl was referred to the Pediatric Nephrology Clinic with a chief complaint of recurrent fevers. In her family history she was the eldest daughter of her family and her parents were non-consanguineous. She was born through natural vaginal delivery and did not have any significant medical history, before the age of 5 years old.

In her past medical history, the patient reported recurrent fevers due to UTIs since 5 years old. During her first febrile episode, she had a 5-day high-grade fever associated with loss of appetite. Her physical examination at that time was unremarkable. Blood tests showed leukocytosis with a shift to the left and urine examination was in favor of pyuria. The urine culture was positive for bacterial growth. Accordingly, the patient was treated for pyelonephritis and got better after 2 days of antibiotic treatment. After 45 days from her first febrile episode, she experienced another episode of fever and loss of appetite. During which, her urine examination was in favor of pyuria. In her blood tests she had leukocytosis with rise in polymorphonuclear leukocytes (PMN) and had an erythrocyte sedimentation rate (ESR) of 95 mm/hr. and a 3+ C-reactive protein. Her kidney function, including blood urea nitrogen (BUN) and serum creatinine levels were normal. Ultrasonography of the kidneys was normal. Voiding cystourethrogram (VCUG) was done due to the recurrent UTI after 2 weeks of her fever, which was also normal. The patient’s periodic fevers were repeated every 45 days during the past 8 years. In the intervals between her febrile episodes, the patient was healthy and laboratory tests were normal. The patient was further evaluated for immunodeficiency and cyclic neutropenia, however her immunoglobulin levels were normal. The patient had multiple referrals to different doctors, including infectious specialists, pediatricians, nephrologists and urologists with the same complaints and her repeated ultrasonography scans and the dimercaptosuccinic acid (DMSA) scans were normal. She was also evaluated for renal stones and microlithiasis which was negative. She was diagnosed as a case of recurrent UTI, although atypical, as she had fever which was in favor of upper urinary tract infections without any evidence of kidney involvement.

During her final visits to the nephrology clinic, the patient was on antibiotics as prophylaxy for UTI and was given Fosfomycin for her fevers. Physical examination was unremarkable. After 20 days from her visit, the patient was admitted to a hospital and was treated for pyelonephritis.

**Fig. 1:**
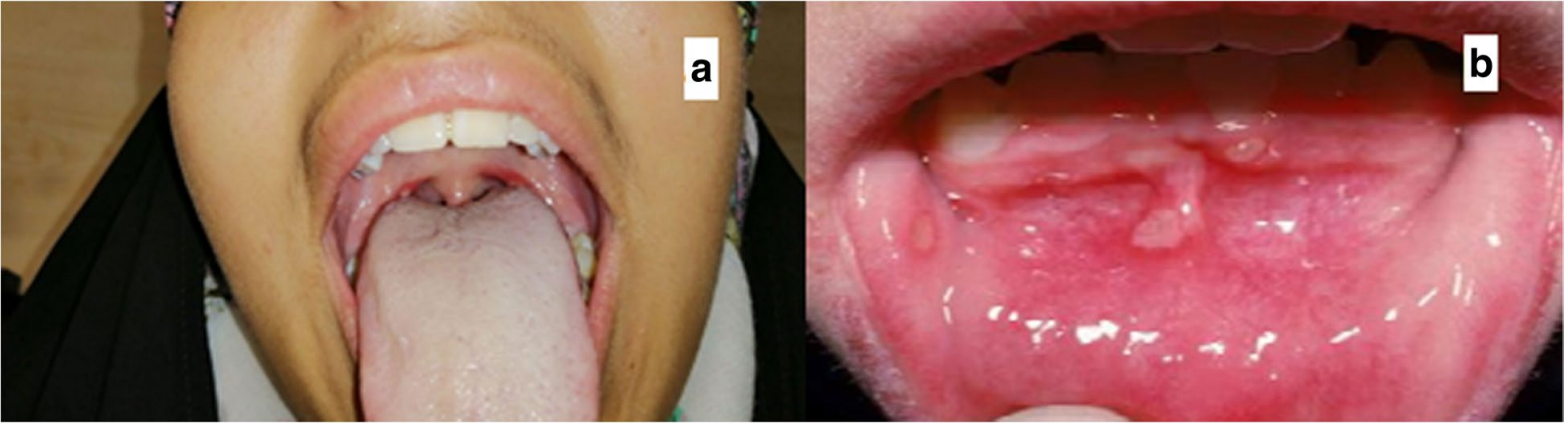
**a** shows exudative bilateral tonsillitis and **b** shows aphthous stomatitis which are visible in the patient’s mouth

During her last visit to the nephrology clinic, she had an axillary temperature of 39.5 degrees. Urine dipstick was also positive for leukocytes and nitrite. Moreover, the patient had an elevated ESR plus pyuria in urine tests and a positive urine culture. During the visit, the patient was ill and mentioned difficulty in swallowing. Accordingly, a thorough physical examination was performed again and 3 cervical lymph nodes, exudative tonsillitis and painful aphthous stomatitis were detected (Fig. 1). Throat cultures were sent and oral penicillin was started. The throat culture was negative. With clinical suspicion for PFAPA, 10 prednisolone forte (50 mg) tablets were given to the patient without any antibiotics. The patient was instructed to take 1.5 tablets (2 mg/kg) in a single dose at time of her fever onset. To date the patient has experienced 5 other febrile episodes, during which all the symptoms have been relieved within 24 h from their onset. Gradually the intervals between the episodes also became longer (3–4 months).

## Discussion and conclusions

Fever is a common unspecific presenting symptom in children. Most fevers resolve before a diagnosis can be made or before any symptoms characteristic of a specific disease develop [3].

Urinary tract infection is the second most common bacterial infection in children, only after otitis media [4], which occurs in 1–3% of girls and usually occurring before the age of five. The three basic forms of UTI are pyelonephritis, cystitis, and asymptomatic bacteriuria. UTI may be suspected based on symptoms or findings in urinalysis or both. Urine culture is necessary for confirmation and appropriate therapy [5].

Recurrent UTI is defined as repeated UTIs with a frequency of 2 or more UTIs in 6 months or 3 or more UTIs within the preceding year, and requires evaluation for any underlying causes such as urological abnormality and urinary dysfunctions. Furthermore, these patients receive antimicrobial prophylaxis [5, 6].

Recurrent fever syndromes are defined as 3 or more episodes of unexplained fever (temperature of 100.4 ° F) during a 6-month period, occurring at least 7 days apart [7, 8].

Periodic fever, aphthous stomatitis, pharyngitis, and adenitis (PFAPA) syndrome is the most common non-genetic form of recurrent fever syndrome in children with an estimated incidence rate of 2.3 in every 1000 children under the age of 5 years old based on one study from Norway, although the exact incidence rate of PFAPA is still unknown [2, 9].

It was first described in 1987 by Marshall et al. [10] and is the most common auto inflammatory disease of childhood. Patients generally feel well between episodes but often suffer considerably during episodes of fever [11]. Currently the diagnosis of PFAPA is based on clinical criteria (Table 1), but these criteria have not been validated in a cohort of patients. Therefore, patients should first be evaluated using clinical findings and genetic studies for other known periodic syndromes before making a diagnosis of PFAPA [8].

**Table 1:** Diagnostic criteria for PFAPA

I. Regularly recurring fevers with an early age of onset (< 5 years of age) II. Constitutional symptoms in the absence of upper respiratory infection with at least 1 of the following clinical signs: a) aphtous stomatitis, b) cervical lymphadenitis, c) pharyngitis III. Complete asymptomatic interval between episodes IV. Normal growth and development	

Tonsillar inflammation is a prominent apparatus among patients with PFAPA. Our patient showed tonsillitis while throat culture was negative for microbial growth. This is due to the fact that tonsillitis, among this group of patients, mostly has an immunological background. Accordingly, previous literature has shown that tonsils from these patients have a high rate of T cell chemokine and proinflammatory cytokine expression rate [12, 13].

In our patient, fevers were not justifiable with a diagnosis of UTI during some of her episodes and the presence of pyuria was justified by the existence of fever [14]. On the other hand, she had an 8 years history of recurrent fevers and was marked as a case of chronic recurrent UTI.

Oral corticosteroids relieve symptoms of PFAPA dramatically. A single dose of prednisone (1–2 mg/kg) or betamethasone (0.1–0.2 mg/kg) given at the onset of an episode can dramatically relieve the fever in a few hours [15]. We used this method for our patient and as a result the patient’s symptoms were relived.

## Conclusion

Our study shows that a patient should never be marked, particularly when the symptom and signs aren’t completely justifying a patient’s condition. The other most important educational massage is the setting at which the patient was first visited, considering if the patient had first been visited by a rheumatologist, the first differential diagnosis for periodic fevers would have probably been PFAPA, thus physicians should consider other diagnosis outside their own specific field of specialty.

## Data Availability

Data sharing not applicable to this article as no datasets were generated or analyzed during the current study.

## Abbreviations

UTI: Urinary tract infection
PMN: Polymorphonuclear leukocytes
ESR: Erythrocyte sedimentation rate
BUN: Blood urea nitrogen
VCUG: Voiding cystourethrogram
DMSA: Dimercaptosuccinic acid
PFAPA: Periodic fever, aphthous stomatitis, pharyngitis, and adenitis
